# Helminths in common eiders (*Somateria mollissima*): Sex, age, and migration have differential effects on parasite loads

**DOI:** 10.1016/j.ijppaw.2019.05.004

**Published:** 2019-05-17

**Authors:** Stine Vestbo, Claus Hindberg, Mark R. Forbes, Mark L. Mallory, Flemming Merkel, Rolanda J. Steenweg, Peter Funch, H. Grant Gilchrist, Gregory J. Robertson, Jennifer F. Provencher

**Affiliations:** a Department of Bioscience, Aarhus University, Aarhus C, Denmark; b Department of Biology, Carleton University, Ottawa, Ontario, Canada; c Department of Biology, Acadia University, Wolfville, Nova Scotia, Canada; d The Greenland Institute of Natural Resources, Nuuk, Greenland; e Department of Biology, Dalhousie University, Nova Scotia, Canada; f Environment and Climate Change Canada, Ottawa, Ontario, Quebec, Canada; g Environment and Climate Change Canada, Mount Pearl, Newfoundland and Labrador, Canada; h Environment and Climate Change Canada, Gatineau, Quebec, Canada

**Keywords:** Arctic parasitology, Body size, Gastrointestinal parasites, Migration, Seabird

## Abstract

In birds, parasites cause detrimental effects to the individual host, including reduced survival and reproductive output. The level of parasitic infection can vary with a range of factors, including migratory status, body size, sex, and age of hosts, or season. Understanding this baseline variation is important in order to identify the effects of external changes such as climate change on the parasitic load and potential impacts to individuals and populations. In this study, we compared the infection level (prevalence, intensity, and abundance) of gastrointestinal parasites in a total of 457 common eiders (*Somateria mollissima*) from four different sampling locations (Belcher Islands, Cape Dorset, West Greenland and Newfoundland), and explored the effects of migration, sex and age on levels of parasitism. Across all samples, eiders were infected with one nematode genus, two acanthocephalan genera, three genera of cestodes, and three trematode genera. Migratory phase and status alone did not explain the observed variation in infection levels; the expectation that post-migratory eiders would be more parasitized than pre-migratory eiders, due to the energetic cost of migration, did not fit our results. No effect of age was detected, whereas effects of sex and body size were only detected for certain parasitic taxa and was inconsistent with location. Since gastrointestinal helminths are trophically-transmitted, future studies of the regional and temporal variation in the diet of eiders and the associated variation and infestation level of intermediate hosts might further explain the observed variation of the parasitic load in eiders in different regions.

## Highlights

• Parasitic infections are understudied for avian populations in the Arctic.
• The helminth infection level in eiders varied greatly between sites and subspecies.
• Migratory phase and status alone did not explain observed helminth variation.
• Inconsistent effects of sex were detected for a few helminth taxa; no effect of age.
• Future studies investigating associations between diet and parasites are needed.

## Graphical abstract

**Figure:**
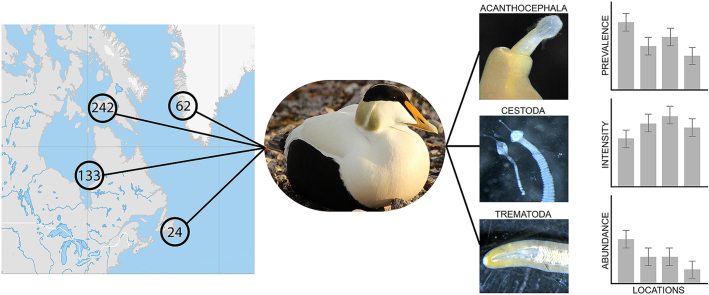

## 1 Introduction

Parasites are important components of ecosystems because they can reduce the impact of grazing herbivores, suppress competitive species, and affect host behavior in order to make them more susceptible to predation (Hatcher et al., 2012; Hudson et al., 2006). Although the presence of parasites can be beneficial to the ecosystem, the individual infected host will likely experience decreased fitness due to either increased predation risk or the energetic costs following infection, such as increased immune response and loss of resources to the parasite (Bonneaud et al., 2003; Hatcher et al., 2012). In birds, parasites can negatively affect several life history traits such as clutch size, number of hatchlings and fledglings (e.g. Marzal et al., 2005; Møller et al., 1990), survival (e.g. Brown et al., 1995), and the quality of both offspring and parental care decreases with increasing parasitism (Knowles et al., 2010). However, in many instances detrimental effects of parasitic infections are undetected (e.g. Bensch et al., 2007; Synek et al., 2013 but see Asghar et al., 2015). As the level of parasitic infection is unknown in many natural bird populations, investigating these fundamental questions is an important first step in understanding how parasites influence the general fitness of individuals and health of populations.

A range of factors can contribute to variation in parasitic infections in birds, including migration strategy. Some studies have shown that migratory birds are more parasitized than non-migratory birds (Koprivnikar and Leung, 2015), which might be explained by increased energetic cost due to migration followed by suppressed immune function (Nebel et al., 2012; Owen and Moore, 2008). Also, Gylfe et al. (2000) showed that stressful conditions such as migration can reactivate infections. In contrast, Arriero and Møller (2008) found that migratory birds have a lower parasitic load of blood parasites compared to non-migratory birds. This result might be explained by migratory escape from parasites or migratory culling of infected individuals, both of which lower the risk of pathogen infection in a migrating population (Hall et al., 2014). However, Gutiérrez et al. (2017) found that habitat diversity rather than migration distance explained the variation in parasite species richness in charadriiform birds.

Intrinsic factors such as host body size, sex and age can also influence the parasitic load in birds. Larger individuals are expected to host a higher parasitic richness since they provide more resources and are likely to be exposed to a wider range of parasites due to their higher food consumption (Kamiya et al., 2014). Males and females often differ in several physiological, morphological, and behavioral traits, and this may result in differences in the parasitic load between sexes (Hasselquist, 2007; McCurdy et al., 1998; Poulin, 1996). One hypothesis is that the sex with the highest variance in reproductive output, most often males, experience more stress during the mating season due to energetic cost of courtship behavior, and hence is more prone to parasitic infections (Hasselquist, 2007). Similarly, the parasitic load might differ between juveniles and adults because of, for instance, age-dependent food sources or increased investment in immune functions in younger birds (Isomursu et al., 2006; Møller et al., 2003).

Understanding baseline levels of parasitic infections, including normal variation, is essential to record any changes or shifts in the parasitic load due to climate change, which is especially important for Arctic species (Davidson et al., 2011). Baseline levels of parasitic infections in birds are also critical in understanding how any future changes in e.g. prey and parasite populations may or may not affect individual and population level health. Furthermore, establishment of baseline infection levels will help identify when and how other stressors might change the effects of infections, e.g. oil spills, which can mimic the effect of anthelminthic drugs (Khan et al., 2011; Thieltges et al., 2006).

The common eider, *Somateria mollissima*, is a species known to host a variety of helminth parasites. Throughout their range they regularly host several species of trematodes, acanthocephalans, cestodes and nematodes (e.g. Galaktionov, 1996; Skirnisson, 2015; Tourangeau et al., 2018). Therefore, the common eider is a useful model species to explore how age, sex, and migration can influence parasitic infection in an avian host species.

Common eider populations consist of six subspecies restricted to the northern hemisphere (Waltho and Coulson, 2015). Three subspecies occur in the western Atlantic: 1) *S. m. borealis*, which has wintering residence in Southwest Greenland or eastern North America (ranging from southern Labrador to Nova Scotia) and breeding residence in East Arctic Canada or West Greenland, and undergoes significant migrations between breeding and wintering areas (Abraham and Finney, 1986; Boertmann et al., 1996; Merkel et al., 2002; Mosbech et al., 2006); 2) *S. m. dresseri*, which winters along the coasts of eastern North America from Newfoundland south to Massachusetts and breeds from southern Labrador to Maine, includes some geographic overlap between breeding and wintering populations so undergoes relatively shorter migrations (Goudie et al., 2000); and 3) *S. m. sedentaria*, a year-round, non-migratory resident in Hudson Bay (Abraham and Finney, 1986, Fig. 1).

**Fig. 1:**
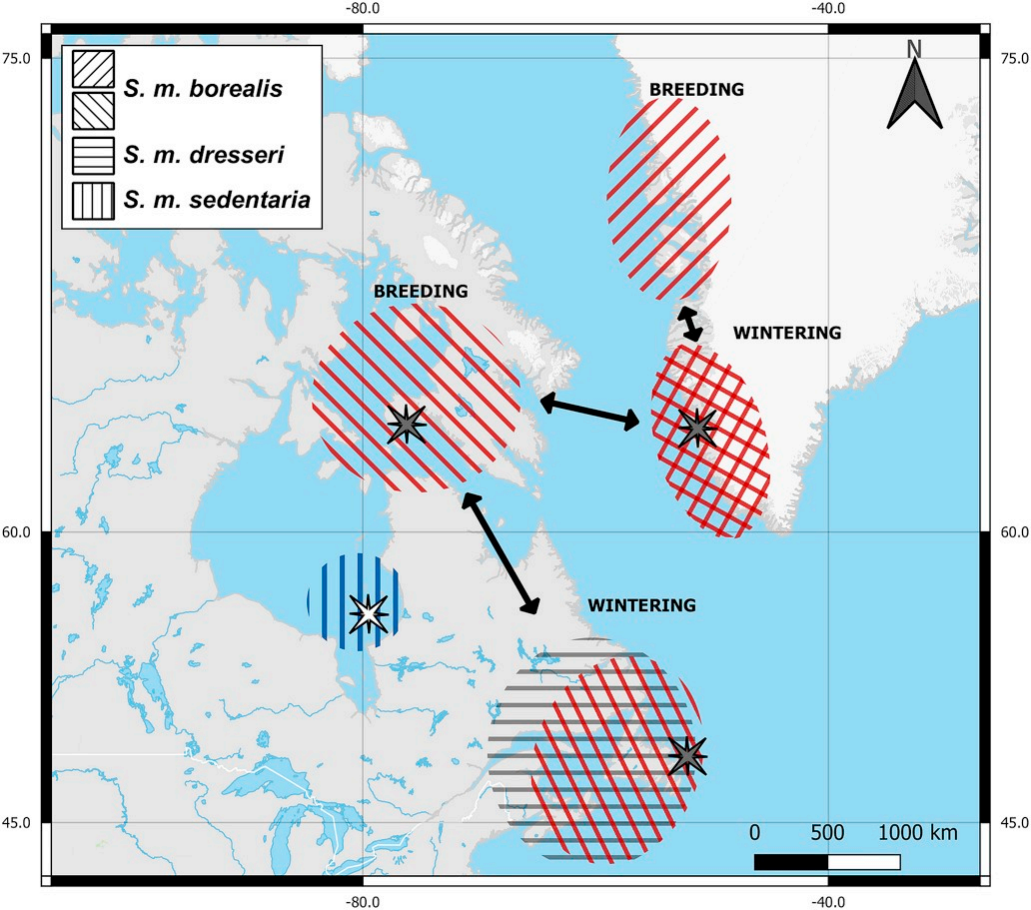
Distributions of breeding and wintering populations of *S. m. borealis*, *S. m. dresseri*, and *S. m. sedentaria* in North America and Greenland. Populations of *S. m. borealis* are wintering in two areas in the pictured region; one in Southwest Greenland and one in East Canada (Newfoundland and Labrador). Individuals wintering in Southwest Greenland migrate to breed in West Greenland or Arctic Canada, whereas individuals wintering in East Canada have breeding areas in Arctic Canada (illustrated by the different direction of the red diagonal lines). For *S. m. dresseri* the breeding and wintering ranges overlap in one area that covers Newfoundland and Labrador as well as the northeastern part of the US, as shown by the grey horizontal lines. The subspecies *S. m. sedentaria* has its year-round residence in the Hudson Bay area as shown by the blue vertical lines. Dark stars mark the sampling locations of eiders in this study, whereas the white star marks the sampling location of eiders by Tourangeau et al. (2018). (For interpretation of the references to colour in this figure legend, the reader is referred to the Web version of this article.)

In eiders, infection levels of gastrointestinal parasites can vary in relation to sex, age and season (Bishop and Threlfall, 1974; Garden et al., 1964; Liat and Pike, 1980; Provencher et al., 2016; Skirnisson, 2015; Thieltges et al., 2006; Thompson, 1985; Tourangeau et al., 2018). Differences in parasitic load between sexes are suggested to be due to different food preferences or physiology in males and females (Provencher et al., 2016), while differences in parasitic load of juveniles and adults have been ascribed to different susceptibility of age groups, acquired immunity in older birds, or age-dependent food preferences (Thieltges et al., 2006). In general, the detrimental effects of parasites in eiders are not well documented. However, Hanssen et al. (2003) found that while incubating, female eiders were mainly unaffected by their parasitic infections, although poor-condition females treated with an anthelminthic drug showed improved return rates the following year. It was suggested that the minute effects of parasites observed might be explained by the female eiders’ reduced food intake during incubation, which likely starves and kills the parasites (Hanssen et al., 2003; Thompson, 1985). Similarly, Provencher et al. (2017) found that anti-parasite treatment increased the probability of nesting in females arriving late or in poor condition to the breeding site. In an experimental infection of eider chicks with the acanthocephalan *Profilicollis minutes*, Hollmén et al. (1999) found that infected chicks gained less weight compared to non-infected chicks. Thus, there is evidence that certain parasites impose energetic costs on eiders, although it appears that they can survive and reproduce with infections. Even though several studies have investigated the parasitic load in eiders, to date, there has been no work on how differing migration strategies may influence helminth parasite burden in common eiders. However, stress metrics and presumed immune function vary with migration strategy in breeding eiders (Mallory et al., 2015).

In this study, we investigated trophically-transmitted gastrointestinal helminths in common eiders from northern and Arctic regions across the Northwest Atlantic. First, we compared the parasite burdens in relation to migratory strategy and migratory phase. Since migration is energetically expensive, eiders on migration are expected to allocate fewer resources to the immune system (Mallory et al., 2015) and as a consequence be more susceptible to parasitic infections (Altizer et al., 2011). Thus, we expected eiders collected in the pre-migration period (eiders sampled in Greenland and Newfoundland in April) to have lower infection levels of parasites as compared with eiders collected on the Arctic breeding grounds (eiders sampled at Cape Dorset in May). Although common eiders are considered less opportunistic feeders than other seabirds, they usually feed on the most abundant prey species (Weslawski et al., 1994) and their infection levels can thus be expected to depend on the distribution and abundance of prey species, suggesting that their infection levels can be expected to vary depending on location (Gutiérrez et al., 2017). Finally, we examined body size, age and sex as possible drivers of infection levels. Since previous studies have shown that the parasitic load in eiders varies among size, sexes and age classes, we expected to find similar, parasite-specific patterns in this study.

## 2 Materials and methods

### 2.1 Capture of birds

We sampled 242 northern common eiders, *S. m. borealis* near Cape Dorset, Nunavut (64°13′N, 76°32′W, Fig. 1), in collaboration with local Inuit hunters from the Cape Dorset Hunter and Trapper Organization (HTO) during the annual spring harvest in May 2011 and 2012 (data from 2011 is already published in Provencher et al. (2016)). The harvest coincides with the eiders' return to the Canadian Arctic after wintering in Greenland and eastern Canada (Fig. 1). All eiders were shot at coastal areas by local hunters with steel shot using 12-gauge shotguns. All samples taken from harvested birds were in compliance with all appropriate Territorial and Federal wildlife research permits (Canadian Wildlife Service, Government of Nunavut). Birds were either frozen or dissected in the community within 36 h of collection. Birds that were frozen were later dissected using the same protocols at the National Wildlife Research Centre (NWRC) in Ottawa or at the Nunavut Arctic College (Provencher et al., 2013). From each bird, the gastro-intestinal tract was removed from esophagus to cloaca and sealed in a polyethylene bag for storage and transport. Post-sampling, the birds dissected in the community were distributed among community members for consumption by the local HTO. All tissues were then excised and deposited at, or shipped to, the NWRC in Ottawa, Canada and put into storage at −40 °C.

In April 2014, 62 eiders of the subspecies *S. m. borealis* drowned as fisheries by-catch in gill nets in Nuuk Fjord (near Qussuk), Greenland (64°45′36″N, 51°00′36″W, Fig. 1), were submitted to the Greenlandic Institute of Natural Resources in Nuuk, Greenland and frozen at −18 °C until dissection. Any eiders showing oil soiling or decomposition were discarded and not dissected. The gastrointestinal tracts, from the gizzard to the cloaca, was removed and sealed in a polyethylene bag and frozen for transport. All samples were shipped to the NWRC and stored at −40 °C.

In April 2016, 24 adult eiders, 16 belonging to *S. m. borealis* and 8 to *S. m. dresseri* were collected under permit (Canadian Wildlife Service) in Newfoundland, Canada (47°'N, 53°53′W, Fig. 1) after flying into light standards at a coastal industry site. Eiders were stored frozen prior to shipment to NWRC. The gastrointestinal tracts, from the gizzard to the cloaca, was removed and sealed in a polyethylene bag for transport and storage and later examined for gastrointestinal helminths.

Body mass (g) was recorded as an index for body size for all individuals. The ducks were sexed by plumage and syrinx morphology (Beer, 1963), and aged by plumage, the length of the bursa of Fabricius and in females by oviduct condition (Baker, 1993; Mather and Esler, 1999). Subspecies were differentiated by bill measurements (Mendall, 1986).

### 2.2 Gastrointestinal parasites

The gastrointestinal tracts were dissected and examined for parasites at the NWRC. The gizzard was separated from the rest of the intestine, and for a subset of eiders sampled in Cape Dorset, Greenland and Newfoundland, the gizzard membrane was removed in order to assess the presence of *Amidostomum* spp. nematodes, which were present in 81 of 87 (93.1%) gizzards examined. However, the presence of *Amidostomum* spp. was not assessed in eiders sampled from the Belcher Islands (Tourangeau et al., 2018, see section 2.3) and hence further analysis of *Amidostomum* spp. nematodes was excluded from this study. Each intestine was sliced open along its entire length without damaging any parasites present. The opened intestine was rinsed with 0.85% saline solution, and any visible helminths were gently removed using forceps or by removing the attachment site and leaving the tissue to soak for a few hours, since this softened the tissue and made it possible to remove the proboscis from the intestinal wall without damaging it. The rinsed solution was further examined under a dissecting microscope. All parasites were counted in each gastrointestinal tract, and the abundance of cestodes was counted by the number of scolices found. However, for *Microsomacanthus* spp. cestodes, the abundance was estimated as 0, 1s, 10s, 100s, or 1000s from aliquots due to the small size of the scolices and the sheer numbers present in many of the samples. This counting method was also applied for the trematodes *Gymnophallus* spp. and *Microphallus* spp. A selection of the helminth specimens were placed in 95% ethanol for further identification. Helminths were stained using acetocarmine, and mounted on microscope slides using Canada balsam. Acanthocephalans, trematodes, and cestodes were identified according to McDonald (1981, 1988) and McLaughlin (2003), respectively.

### 2.3 Statistical analysis

Four metrics for quantifying parasite numbers were considered: prevalence, mean and median intensity, and mean abundance. Prevalence was calculated as the proportion of infected birds in the sample. Mean and median intensity was calculated as the average and median number of parasites per infected bird, respectively (excluding uninfected individuals). Mean abundance was calculated as the average number of parasites per bird, thus including both infected and uninfected individuals. The confidence intervals for the intensity metrics was calculated using bootstrap methods, as the number of infected individuals in some of the groups was less than 30 (Rózsa et al., 2000). Due to the large numbers of parasites for some of the platyhelminth taxa (*Microsomacanthus*, *Microphallus* and *Gymnophallus*) the abundances of these were scored as 0, 1s, 10s, 100s, and 1000s, hence only prevalence could be calculated for these taxa.

All statistical analyses were carried out in R, version 3.4.3 (R Core Team, 2017) and a significance level of 0.05 was applied for all statistical tests. Correlations between host body size and parasite richness and abundance for each location (Cape Dorset, Greenland and Newfoundland) were assessed using Kendall rank correlation coefficient due to the non-normality of data. Parasite prevalences were compared between locations and subspecies using Fisher's exact test, as recommended by Rózsa et al. (2000). For the parasites belonging to the cestode genus *Lateriporus*, the two acanthocephalan genera *Profilicollis* and *Corynosoma*, and the trematode genus *Notocotylus*, mean abundance and intensity metrics were compared using bootstrap *t*-tests with 10,000 samples using package *Hmisc* v4.1-1 (Harrell Jr, 2018), because of the skewness in the data (Rózsa et al., 2000) and medians were compared with Mood's median test using package *RVAideMemoire* (Hervé, 2018). The infection levels of *Microsomacanthus* spp., *Microphallus* spp., and *Gymnophallus* spp. were compared between groups using χ^2^ tests. The effect of host sex on prevalence and intensity was tested using generalized linear models (GLMs). For eiders caught in Greenland, the effect of age was also tested using GLMs. For parasite prevalences, GLMs with binomial distribution and logit link function were applied, and for intensity metrics, negative binomial GLMs were applied on logarithmic transformed data.

Data from *S. m. borealis* caught in Cape Dorset in 2011 reported by Provencher et al. (2016) were included in the analysis. However, before combining the 2011 and 2012 datasets, interannual variation in the prevalence, intensity and abundance of the parasitic taxa was explored using above analyses. In all comparisons, only few significant differences were found: the prevalence, intensity, and abundance of *Fimbriarioides* sp. increased from 2011 to 2012 (*p* < 0.0001, *p =* 0.03 and *p* = 0.043, respectively), the intensity of *Lateriporus* sp. decreased from 2011 to 2012 (*p* = 0.021), and the prevalence of *Corynosoma* sp. was higher in 2012 compared to 2011 (*p* < 0.0001). Due to these few significant findings, the datasets from the birds caught in Cape Dorset in 2011 and 2012 were combined. This decision is also supported by the findings of Tourangeau et al. (2018) where interannual variation for a period of three years was evident for only one parasitic taxon, *Profilicollis* sp., in one metric (abundance). The results reported in this study were compared to results from Tourangeau et al. (2018), reporting parasite burdens from 133 common eiders of the non-migratory subspecies *S. m. sedentaria* from Belcher Islands, Canada.

## 3 Results

### 3.1 Sex ratio and parasitic taxa

**Fig. 2:**
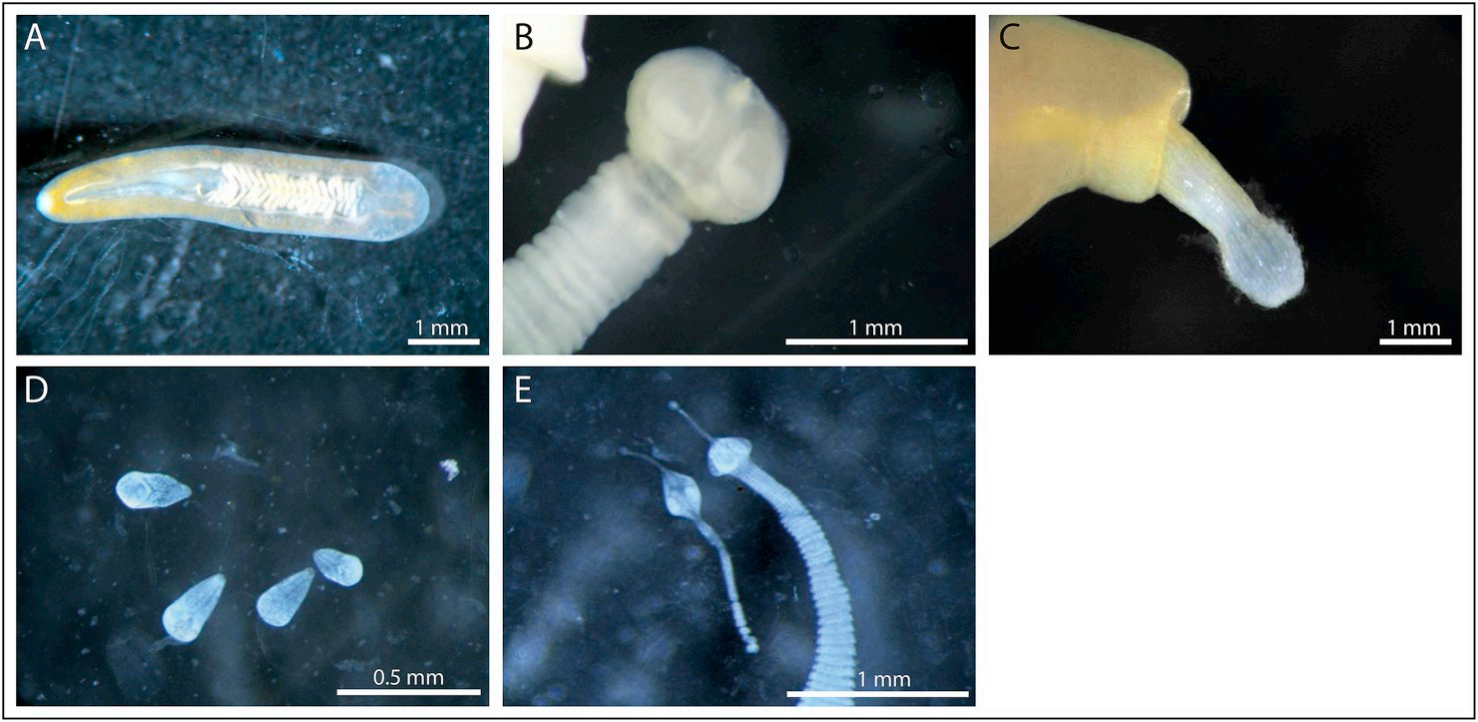
Examples of gastrointestinal parasites retrieved from common eiders in this study. (A) The trematode *Notocotylus* sp., (B) the cestode *Lateriporus* sp., (C) the acanthocephalan, *Profilicollis* sp. (D) microphallid trematodes, and (E) *Microsomacanthus* spp. cestodes.

The percentage of female eiders in samples varied from 42% to 69% depending on location and subspecies (Table 1). In Newfoundland and Cape Dorset, only adult eiders were retrieved, whereas the percentage of adults was 92% in Greenlandic eiders. Across all 328 samples, nine genera of gastrointestinal parasites belonging to three phyla (Acanthocephala, Nematoda and Platyhelminthes) were found in the eiders. The eiders sampled in Greenland were infected with two genera of cestodes, *Lateriporus* and *Microsomacanthus*, one acanthocephalan genus, *Profilicollis*, one nematode genus, *Amidostomum*, and two trematode genera, *Microphallus* and *Gymnophallus* (Fig. 2). These six genera were also found in the *S. m. borealis* from Newfoundland, in addition to the cestode genus *Fimbriarioides* and trematode genus *Notocotylus* (Fig. 2). The *S. m. dresseri* from Newfoundland hosted all mentioned parasitic genera, except *Notocotylus*, and hosted an additional acanthocephalan genus, *Corynosoma*.

**Table 1:** Prevalence, intensity and abundance of gastrointestinal helminth parasites found in common eiders residing in Greenland and Canada. Mean intensity describes the average number of parasites individuals per infected bird, whereas mean abundance describes the average number of parasites in the whole sample. Confidence intervals are given in parentheses for all parameters.

Common eider subspecies	*S. m. borealis*	*S. m. borealis*	*S. m. dresseri*	*S. m. borealis*
Year, month	2014, April	2016, April	2016, April	2011, 2012, May
Location	SW Greenland	Newfoundland	Newfoundland	Cape Dorset
n	62	16	8	242
% female	47	69	62	42
% adult	92	100	100	100
**Cestoda**				
*Lateriporus* sp.				
Prevalence	0.24 (0.15–0.36)	0.44 (0.23–0.67)	0.375 (0.14–0.69)	0.75 (0.69–0.80)
Mean intensity	3.8 (1.67–6.47)	8.57 (4.43–14.6)	10 (1–20)	23.9 (18.5–30.2)
Median intensity	2.0 (1.0–16.85)	6.0 (2.3–23.6)	9.0 (1.4–19.5)	11 (1–116)
Mean abundance	0.92 (0.31–0.77)	3.75 (1.13–7.69)	3.75 (0.13–8.75)	17.9 (13.9–22.9)
*Fimbriarioides* sp.				
Prevalence	0	0.13 (0.035–0.36)	0.25 (0.07–0.59)	0.12 (0.08–0.17)
Mean intensity	–	1 (1–1)	1 (1–1)	13.3 (6.0–22.2)
Median intensity	–	1 (1–1)	1 (1–1)	2.0 (1.0–65.8)
Mean abundance	–	0.13 (0.00–0.31)	0.25 (0–0.63)	1.60 (0.59–2.84)
*Microsomacanthus* spp.				
Prevalence	0.62 (0.50–0.73)	1 (0.81–1)	0.75 (0.41–0.93)	0.88 (0.84–0.92)
**Acanthocephala**				
*Profilicollis* sp.				
Prevalence	0.064 (0.025–0.15)	1 (0.81–1)	1 (0.68–1)	0.73 (0.67–0.78)
Mean intensity	4.75 (2.00–8.25)	19.6 (12.0–27.9)	44.6 (2.0–125.5)	30.9 (23.7–39.5)
Median intensity	4.0 (1.15–9.63)	14.5 (1.75–51.25)	2.0 (1.18–271.3)	9.5 (1.0–183.6)
Mean abundance	0.31 (0.016–0.71)	19.6 (11.56–28.94)	44.62 (2.0–125.5)	22.5 (16.5–28.8)
*Corynosoma* sp.				
Prevalence	0	0	0.13 (0.006–0.47)	0.058 (0.035–0.095)
Mean intensity	–	–	2 (na-na)	2.2 (1.43–3.29)
Median intensity	–	–	2 (2–2)	1.5 (1.0–6.7)
Mean abundance	–	–	0.25 (0–0.75)	0.13 (0.05–0.23)
**Trematoda**				
Notocotylus sp.				
Prevalence	0	0.19 (0.066–0.43)	0	0.012 (0.004–0.036)
Mean intensity	–	1.33 (1.00–2.00)	–	1 (1–1)
Median intensity	–	1.0 (1.0–1.95)	–	1 (1–1)
Mean abundance	–	0.25 (0.00–0.56)	–	0.012 (0–0.025)
Microphallus spp.				
Prevalence	0.71 (0.59–0.81)	1 (0.81–1)	0.88 (0.53–0.99)	na
Gymnophallus spp.				
Prevalence	0.94 (0.85–0.97)	0.13 (0.035–0.36)	0.13 (0.006–0.47)	na

The prevalence, mean and median intensity, as well as mean abundance of the gastrointestinal parasites in the common eiders are summarized in Table 1. Where appropriate, the prevalences, intensities, and abundances found in this study were compared to data from a previous study (see section 2.3).

### 3.2 Variation between pre-, post- and non-migratory eiders

**Fig. 3:**
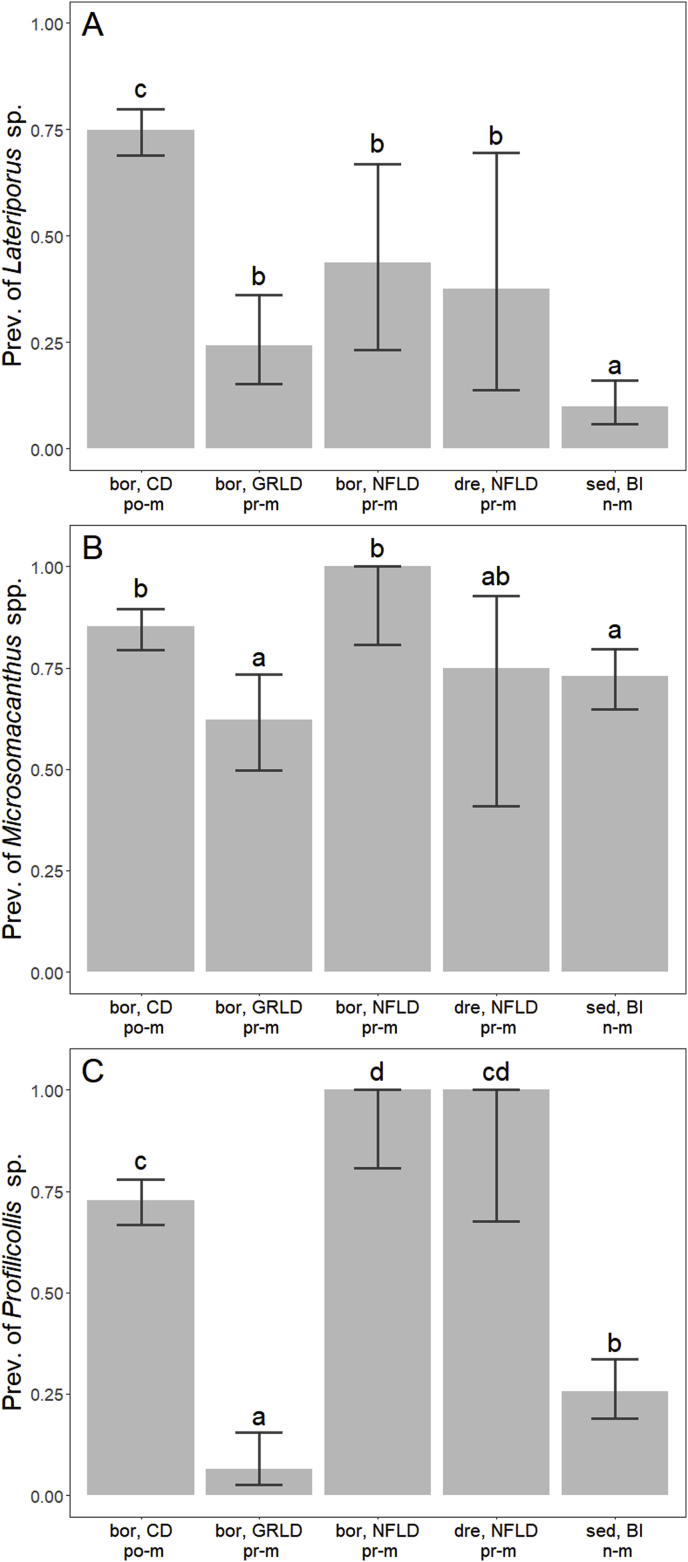
Prevalences of the cestodes *Lateriporus* sp. (A) and *Microsomacanthus* spp. (B), and the acanthocephalan *Profilicollis* sp. (C) in common eiders. Abbreviations: bor, CD = *S. m. borealis*, Cape Dorset; bor, GRLD = *S. m. borealis,* Greenland; bor, NFLD = *S. m. borealis,* Newfoundland; dre, NFLD = *S. m. dresseri,* Newfoundland; sed, BI = *S. m. sedentaria* [data published in Tourangeau et al. (2018)]. n-m = non-migratory, po-m = post-migratory, pr-m = pre-migratory. Letters describe significant differences between groups: if two groups share a letter, there is no significant difference in their prevalences.

**Fig. 4:**
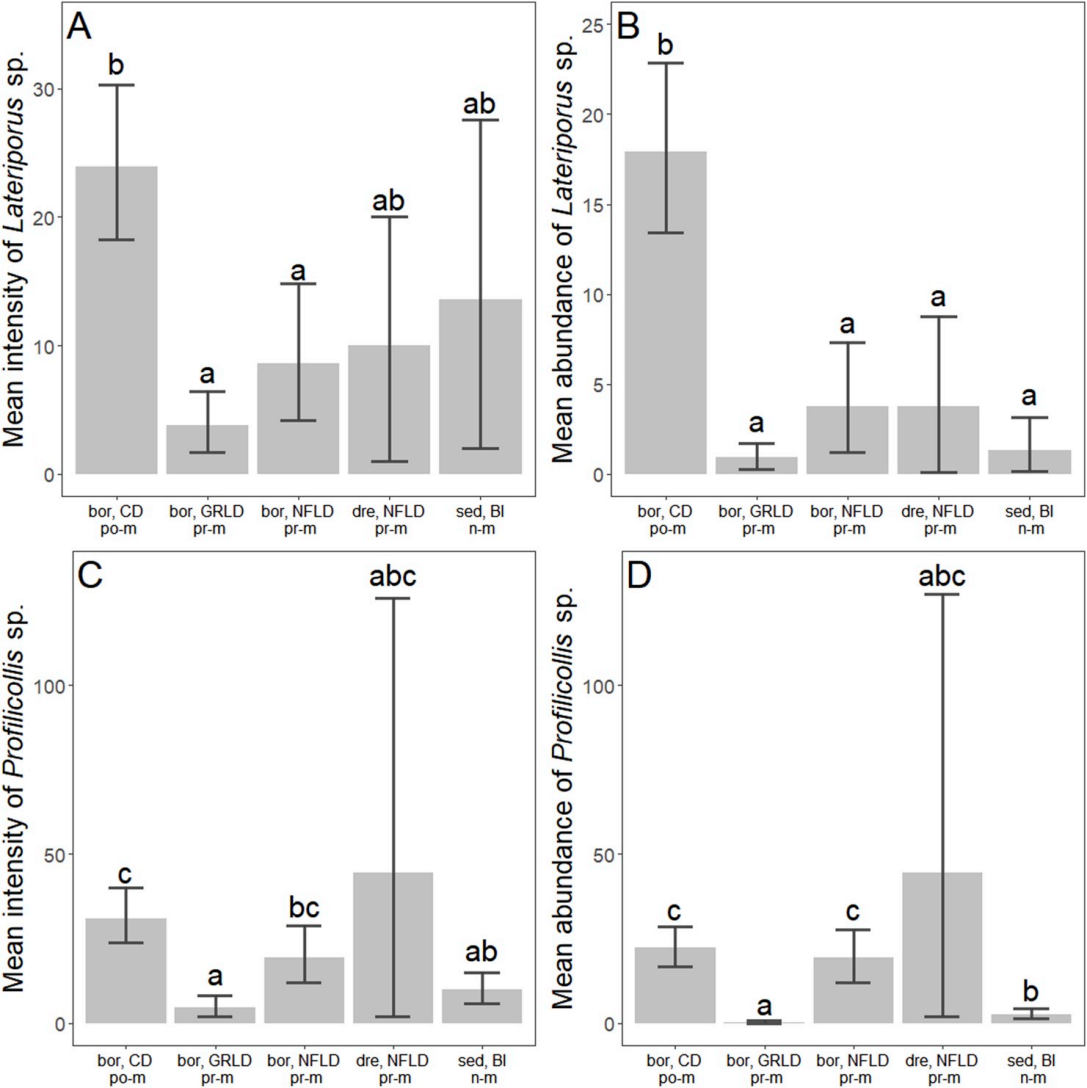
Mean intensity and mean abundance of *Lateriporus* sp. (A, C) and *Profilicollis* sp. (C, D) for common eiders. Abbreviations: bor, CD = *S. m. borealis*, Cape Dorset; bor, GRLD = *S. m. borealis,* Greenland; bor, NFLD = *S. m. borealis,* Newfoundland; dre, NFLD = *S. m. dresseri,* Newfoundland; sed, BI = *S. m. sedentaria* [data published in Tourangeau et al. (2018)]. n-m = non-migratory, po-m = post-migratory, pr-m = pre-migratory. Letters describe significant differences between groups: if two groups share a letter, there is no significant difference between their means.

Post-migratory eiders from Cape Dorset were generally more infected with *Lateriporus* sp. cestodes compared to non- and pre-migratory eiders, and both prevalence and abundance was significantly higher (Table 2, Fig. 3A, Fig. 4A and B). Also, the mean intensity was higher for post-migratory eiders compared to pre-migratory *S. m. borealis* from Greenland and Newfoundland (Table 2, Fig. 3, Fig. 4A, B). Post-migratory eiders had significantly higher median intensity compared to non-migratory eiders from Belcher Islands and pre-migratory eiders sampled from Greenland. Pre-migratory *S. m. borealis* from Newfoundland also showed higher median intensity than pre-migratory eiders sampled from Greenland (Table 2), and non-migratory eiders had significantly lower prevalence of *Lateriporus* sp. compared to the four groups of migratory eiders (Table 2, Fig. 3A).

**Table 2:** Overview of *p*-values from statistical tests comparing the prevalences (Prev.), mean intensities (Mean int.), median intensities (Med int.) and mean abundances (Abund.) of three helminth taxa, which were present in all locations, in common eiders. Only prevalence could be compared for *Microsomacanthus* spp. due to categorical data. Asterisks mark the level of significance, where * = *p* > 0.05, ** = *p* > 0.01, and *** = *p* > 0.001.

	*Lateriporus* sp.				*Profilicollis* sp.				*Microsomacanthus* spp.
	Prev.	Mean int.	Med int.	Abund.	Prev.	Mean int.	Med int.	Abund.	Prev.
	**Belcher Islands** (*S. m. sedentaria*)								
**Cape Dorset** (*S. m. borealis*)	***	0.26	*	***	***	***	0.13	***	***
**Newfoundland** (*S. m. borealis*)	**	0.57	0.06	0.26	***	0.07	0.069	**	*
**Newfoundland** (*S. m. dresseri*)	*	0.43	0.55	0.40	***	0.41	0.70	0.42	1
**Greenland** (*S. m. borealis*)	*	0.28	0.69	0.67	**	0.10	1	*	0.18
	**Cape Dorset** (*S. m. borealis*)								
**Newfoundland** (*S. m. borealis*)	*	***	0.12	***	*	0.07	0.43	0.59	0.23
**Newfoundland** (*S. m. dresseri*)	*	*	1	***	0.11	0.61	0.28	0.61	0.35
**Greenland** (*S. m. borealis*)	***	***	**	***	***	***	0.62	***	**
	**Newfoundland** (*S. m. borealis*)								
**Newfoundland** (*S. m. dresseri*)	1	0.81	1	0.99	1	0.60	0.19	0.59	0.70
**Greenland** (*S. m. borealis*)	0.13	0.28	*	0.21	***	*	0.08	**	**
	**Newfoundland** (*S. m. dresseri*)								
**Greenland** (*S. m. borealis*)	0.42	0.39	0.25	0.35	***	0.42	0.55	0.80	0.10

**Fig. 5:**
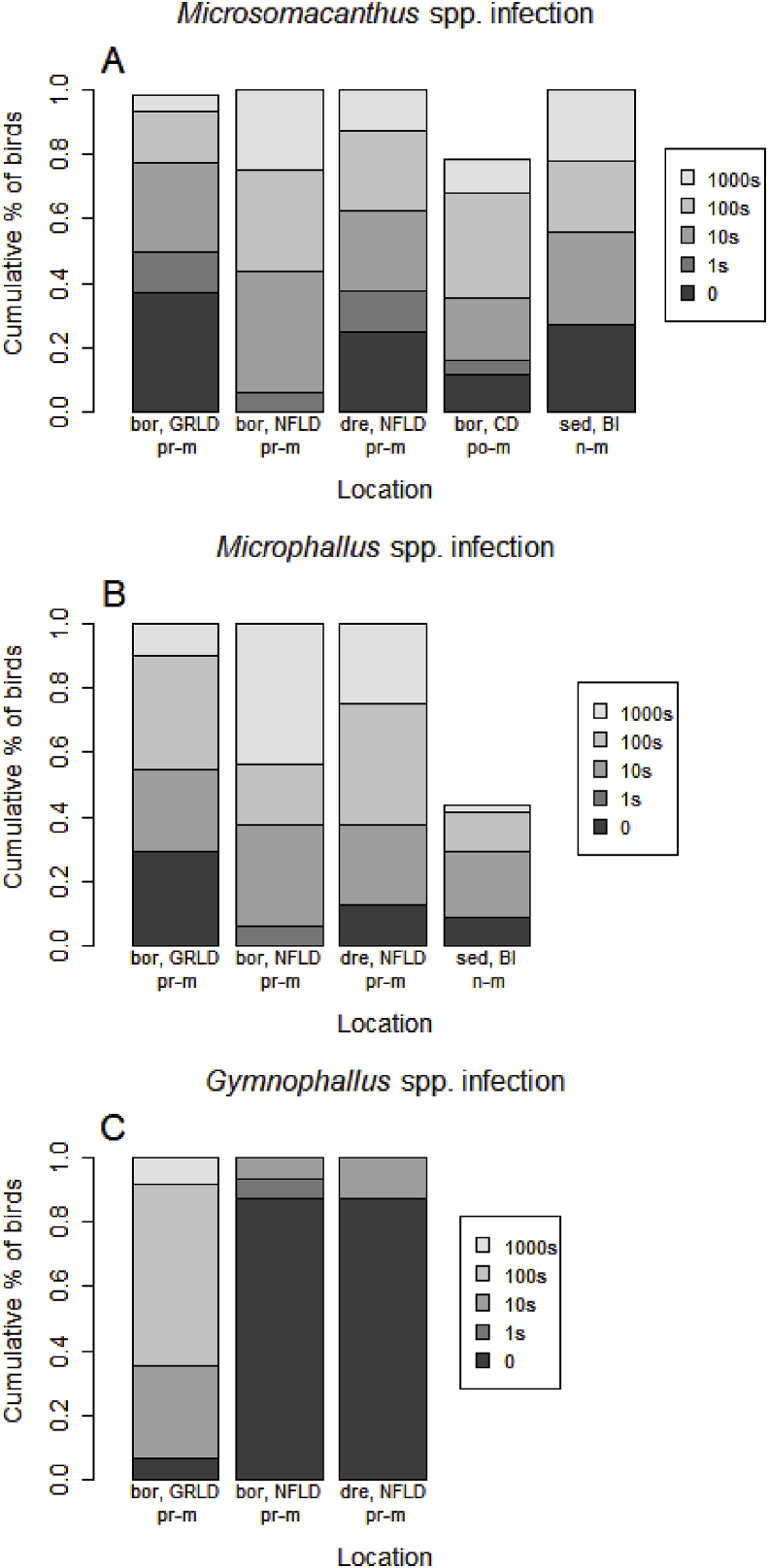
Cumulative percentages of birds infected with (A) *Microsomacanthus* spp., (B) *Microphallus* spp. and (C) *Gymnophallus* spp. from five different locations. Legend denotes the five infection levels: 0, 1s, 10s, 100s, and 1000s of parasite individuals within a single host. Abbreviations: bor, GRLD = *S. m. borealis,* Greenland; bor, NFLD = *S. m. borealis,* Newfoundland; dre, NFLD = *S. m. dresseri,* Newfoundland; bor, CD = *S. m. borealis*, Cape Dorset; sed, BI = *S. m. sedentaria,* Belcher Islands [data published in (Tourangeau et al., 2018)]. n-m = non-migratory, po-m = post-migratory, pr-m = pre-migratory.

Higher prevalences of *Microsomacanthus* spp. cestodes were found in pre-migratory *S. m. borealis* from Newfoundland and post-migratory eiders compared to pre-migratory eiders from Greenland and non-migratory eiders (Table 2, Fig. 3B). Both post-migratory eiders and non-migratory eiders had a higher proportion of individuals highly infected with *Microsomacanthus* spp. compared to pre-migratory eiders from Greenland (Fig. 5A, χ^2^ = 26.5 and χ^2^ = 27, respectively, *p* < 0.0001 for both), and non-migratory eiders were less infected compared to post-migratory eiders (Fig. 5A, χ^2^ = 25.2, *p* < 0.0001).

The prevalence of the acanthocephalan *Profilicollis* sp. in pre-migratory eiders from Greenland was significantly lower than in any of the other four groups (Table 2, Fig. 3C). Non-migratory eiders also showed a low *Profilicollis* sp. prevalence compared to both post-migratory eiders from Cape Dorset and pre-migratory eiders from Newfoundland, and post-migratory eiders had a lower prevalence than pre-migratory *S. m. borealis* from Newfoundland (Table 2, Fig. 3C). The mean intensity of *Profilicollis* sp. was higher in post-migratory eiders and pre-migratory *S. m. borealis* from Newfoundland than in pre-migratory eiders from Greenland, and non-migratory eiders had a lower mean intensity than post-migratory eiders (Table 2, Fig. 4C). The median intensity of *Profilicollis* sp. did not differ between any of the groups (Table 2). Pre-migratory eiders sampled from Greenland had the lowest mean abundance of *Profilicollis* sp., followed by non-migratory eiders, which was surpassed by post-migratory eiders and pre-migratory *S. m. borealis* from Newfoundland (Table 2, Fig. 4D). Thus, generally, post-migratory eiders sampled from Cape Dorset and pre-migratory eiders sampled from Newfoundland tended to be more infected by *Profilicollis* sp. than pre-migratory eiders sampled from Greenland and non-migratory eiders from Belcher Islands.

The prevalence of *Microphallus* spp. trematodes did not differ between pre-migratory and non-migratory eiders (data for *Microphallus* spp. were not available for post-migratory eiders), although a larger proportion of pre-migratory *S. m. borealis* from Newfoundland had higher infection levels of *Microphallus* spp. compared to pre-migratory eiders from Greenland and non-migratory eiders (χ^2^ = 21.8 and χ^2^ = 18.6, respectively, *p* < 0.001 for both, Fig. 5B).

There were no differences between eider groups in the prevalence, intensities or abundances of *Fimbriarioides* sp. (all *p* values > 0.05) except that the mean intensity of this cestode was higher for post-migratory eiders than pre-migratory eiders sampled from Newfoundland (*p* < 0.04 for both). The trematode *Notocotylus* sp. was only found in *S. m. borealis* from Newfoundland and Cape Dorset, and the prevalence was higher in pre-migratory Newfoundland eiders compared to post-migratory Cape Dorset eiders (*p* = 0.004). There were no significant differences in the intensity or abundance of *Notocotylus* sp. between the two groups (all *p* values > 0.9). Also, there were no differences between *S. m. dresseri* from Newfoundland and post-migratory eiders in the prevalence, intensities, and abundance of the acanthocephalan *Corynosoma* sp. (all *p* values > 0.1).

The two subspecies, *S. m. borealis* and *S. m. dresseri*, a long and short distance migrant, respectively, sampled from Newfoundland did not differ in their parasitic load for any of the parasitic helminths (all *p* values > 0.1).

### 3.3 Variation within pre-migratory (wintering) eiders

The two groups of pre-migratory eiders sampled in Greenland and Newfoundland, respectively, differed in their parasitic load. *S. m. borealis* from Newfoundland had median intensity of *Lateriporus* sp., mean intensity and abundance of *Profilicollis* sp., and prevalence of *Microsomacanthus* spp., which were higher compared to eiders sampled in Greenland (Table 2, Fig. 3, Fig. 4). A larger proportion of *S. m. borealis* sampled in Newfoundland were highly infected with *Microsomacanthus* spp. compared to eiders sampled in Greenland (Fig. 5A, χ^2^ = 14, *p* = 0.007), but there was no difference in the level of infection between both of these and *S. m. dresseri* from Newfoundland (χ^2^ = 1.34, *p* = 0.85 and χ^2^ = 4.97, *p* = 0.29, respectively). The prevalence of *Profilicollis* sp. was higher in both subspecies from Newfoundland compared to Greenland (Table 2, Fig. 3C).

The prevalence of *Microphallus* spp. trematodes was higher in the eiders from Greenland compared to *S. m. borealis* from Newfoundland (*p =* 0.016), but a larger proportion of the *S. m. borealis* from Newfoundland had higher infection levels of *Microphallus* spp. compared to *S. m. borealis* from Greenland (χ^2^ = 21.8, *p* < 0.001, Fig. 5B). For *Gymnophallus* spp. trematodes, the eiders from Greenland showed a higher prevalence than both groups of Newfoundland eiders (*p* < 0.0001 for both), and in contrast to the findings for *Microphallus* spp., a larger proportion of eiders from Greenland had higher infection levels compared to *S. m. borealis* from Newfoundland (χ^2^ = 53.1, *p* < 0.0001, Fig. 5C).

### 3.4 Body size, sex and age effects

Parasite richness was weakly positively correlated (τ = 0.26) with body size for eiders sampled in Cape Dorset (*p* < 0.0001), but weakly negatively correlated (τ = −0.24) with body size for eiders sampled in Greenland (*p* = 0.02). There was no correlation between parasite richness and body size for eiders sampled in Newfoundland. Parasite abundances were positively correlated with host body size for the cestodes *Lateriporus* sp. and *Fimbriarioides* sp. as well as for the acanthocephalan *Corynosoma* sp., (all *p* < 0.002) for eiders sampled in Cape Dorset, although correlation coefficients were low (all τ > 0.25). There were no correlations between host body size and parasite abundances for eiders sampled in Greenland or Newfoundland.

There was no effect of sex on prevalence or intensity of *Lateriporus* sp., *Microsomacanthus* spp., *Profilicollis* sp., *Corynosoma* sp., or *Gymnophallus* spp. in any of the eiders sampled from Greenland or Newfoundland (too few data to test *Fimbriarioides* sp. and *Notocotylus* sp. intensities). Sex had an effect on *Microphallus* spp. prevalence in eiders from Greenland, where females were more infected than males (z_1,60_ = −2.81, *p* = 0.005). Male eiders from Cape Dorset had a higher prevalence of *Fimbriarioides* sp. and *Lateriporus* sp. compared to females (z_1,60_ = 2.35, *p* = 0.02 and z_1,240_ = −3.12, *p* = 0.002, respectively). There was no effect of age on parasite prevalence and intensity in eiders from Greenland.

## 4 Discussion

### 4.1 General infection levels

In general, the infection levels of eiders found in this study corresponds well with the range of levels observed in previous studies. Provencher et al. (2017) investigated the parasitic load of gastrointestinal parasites in a few, untreated male eiders (*S. m. borealis*), caught in June at Southampton Island, Nunavut when they were returning to the breeding area. They found that abundances and intensities of gastrointestinal helminths were similar to the load observed for the eiders from Cape Dorset and Newfoundland presented here. Khan et al. (2011) found prevalence and intensity of *Profilicollis botulus* from eiders caught during winter in Newfoundland that approximated what we observed for *Profilicollis* sp. in Newfoundland eiders, as did Thieltges et al. (2006) for eiders (*S. m. mollissima*) wintering in the northern Wadden Sea. Skirnisson (2015) investigated the prevalence and intensity of helminths in *S. m. borealis* sampled four times within the same year in Iceland, and reported numbers consistent with our results for eiders from Cape Dorset and Newfoundland, but prevalences of *Gymnophallus* spp. and *Microphallus* spp. were closer to values we observed in eiders sampled from Greenland.

### 4.2 Variation in infection levels in relation to migratory phase and strategy

The parasitic burden of migratory eiders (Greenland, Cape Dorset, and Newfoundland) reported here were generally greater than the parasite infection levels of the non-migratory subspecies from Belcher Islands reported by Tourangeau et al. (2018), but the patterns observed did not follow the migratory/non-migratory dichotomy as predicted. Due to the energetic costs of migration, we expected the post-migratory eiders sampled at Cape Dorset to have higher parasitic loads compared to all other eider groups (pre- and non-migratory) included in this study. Although the post-migratory eiders had significantly higher prevalences, intensities, and abundances of certain parasites compared to the eiders sampled at Belcher Islands (non-migratory) and Greenland (pre-migratory), the parasitic load of eiders sampled in Newfoundland (pre-migratory) resembled that of the post-migratory eiders. These findings suggest that the energetic cost of one month of migration do not increase the infection levels of gastrointestinal helminths in common eiders, and that the distribution of different parasitic taxa can vary across the host distribution depending on habitat.

The life span of gastrointestinal parasites varies across taxa, and a species such as the acanthocephalan *Profilicollis botulus* can live in the final host for two to three months (Thompson, 1985). For adult *Lateriporus* cestodes, the life span is around one month, whereas for the smaller *Microsomacanthus* cestodes it is around two weeks (Denny, 1969). Microphallid trematodes usually survive in the final host for 6–14 days (Galaktionov et al., 2012). Thus, individuals of *Profilicollis* sp. and *Lateriporus* sp. could have been carried with the birds from the wintering areas to the breeding area. Since eiders of the subspecies *S. m. borealis* move at about 60 km per day during spring migration, they likely feed along the way (Mosbech et al., 2006), which suggest that the post-migratory eiders examined in this study could have acquired additional infections during migration. However, it should be noted that *S. m. borealis* migrating from Newfoundland to Arctic Canada during spring follow a coastal migration route, and hence have continuous opportunity to forage, whereas the eiders migrating from Greenland to Arctic Canada spend some part of the journey over sea when crossing the Davis Strait and would thus have fewer opportunities to feed and acquire infections during migration (Waltho and Coulson, 2015). The post-migratory birds sampled in Cape Dorset could be coming mainly from wintering areas in Newfoundland, which would mean that one month of migration does not change their infection levels, even though the eiders have the opportunity to forage during migration and certain parasites are able to survive in the digestive tracts until the breeding area is reached. The post-migratory birds could also be coming mainly from Greenland, and since they are not expected to feed as much during migration, the increased infection levels in the post-migratory eiders would be due to an assumed change in the benthic community between the two sites. Finally, the post-migratory birds could consist of a mix of birds coming from Greenland and Canada, which would mean that the high levels of helminth infections in the post-migratory eiders could be due to several factors. Thus, the results from our study seem to support the findings from Gutiérrez et al. (2017) that habitat rather than migration influence the infection levels of helminths in birds.

### 4.3 Regional variation in infections

The differences observed within the pre-migratory eiders could be due to differences in prey diversity and variability at the two wintering areas, Greenland and Newfoundland. The continental shelf waters of Labrador and Newfoundland have higher primary production compared to the waters off Southwest Greenland (O'Reilly and Sherman, 2016), thus the benthic invertebrate community might be richer and more abundant in the Newfoundland waters compared to Greenland, supporting a larger diversity and abundance of helminth parasites. Indeed, differences in the diversity and abundances of potential prey species could be a direct explanation of the observed variation in the parasitic load of eiders between locations. For example, Skirnisson (2015) showed that the intra-annual variation of gastrointestinal helminths in common eiders from Iceland varied with diet or proportion of intermediate hosts consumed. Although the full life cycles and thereby the intermediate host species of many gastrointestinal helminths are unknown, some general information is available that can be used to explain variation in infections.

Merkel et al. (2007) found that common eiders wintering in Southwest Greenland were feeding primarily on bivalves and annelids, which could explain why the Greenlandic eiders investigated here are less parasitized by cestodes and acanthocephalans, which use crustaceans as intermediate hosts (Galaktionov, 1996). Instead, the eiders caught in Greenland were more heavily parasitized by *Gymnophallus* spp. trematodes, which use bivalves as their intermediate hosts, e.g. *G. somateriae* is commonly found as metacercaria in *Hiatella arctica* (previously *H. byssifera*) (Petersen, 1984; Andersen and Hartmann, 1992), *Limecola (=Macoma) balthica* and *Cerastoderma edule* (Lauckner, 1971; Pekkarinen, 1987), and *G. bursicola* have been reported in both common eiders and blue mussels, *Mytilus edulis* (Galaktionov et al., 2015; Skirnisson, 2015). *H. arctica*, *L. balthica*, and *M. edulis* are all important food items for common eiders during winter in Southwest Greenland (Merkel et al., 2007). Decapods such as *Hemigrapsus oregonensis*, *Carcinus maenas*, *Hyas araneus*, and *Pagurus pubescens* are intermediate hosts for *Profilicollis botulus* (synonym *Polymorphus botulus*), which is also known from common eiders (Ching, 1989), but among these only *H. araneus* has been found in the digestive tract of eiders wintering in Greenland (Merkel et al., 2007), which might explain the minor infection level of *Profilicollis* sp. in the eiders sampled in Greenland. While *P. botulus* uses decapods as intermediate hosts, cestodes such as *Lateriporus* sp. and *Microsomacanthus* spp. utilize amphipods as their intermediate hosts (McDonald, 1969). Merkel et al. (2007) found several species of amphipods in the esophagus and proventriculus of eiders wintering in Greenland, which corroborates the high infection level of *Microsomacanthus* cestodes found in this study, but not the low infections levels of *Lateriporus* sp. *Littorina* spp. snails are the intermediate hosts for microphallid trematodes, and the infection intensity of *Microphallus* spp. in eiders varies with the intake of *Littorina* spp. (Skirnisson, 2015). In Southwest Greenland, eiders feed on *Littorina* snails although they only constitute a minor part of the diet (Merkel et al., 2007). However, *Microphallus pseudopygmaeus*, which is suggested to mainly use common eiders as its final host, infects several gastropod intermediate hosts, including *Littorina* spp., *Margarites helicinus*, *Onoba aculeus* and *Falsicingula athera* (Galaktionov et al., 2004, 2012). Since Merkel et al. (2007) found that *M. helicinus* was the most common gastropod consumed by eiders in Southwest Greenland, this could be an explanation for the high infection levels of *Microphallus* spp. in eiders sampled from Greenland in our study.

Goudie and Ankney (1986) found that common eiders wintering in Newfoundland foraged mainly on the sea urchin *Strongylocentrotus droebachiensis* and blue mussels, which are also the main prey items for eiders wintering in the Gulf of St. Lawrence (Guillemette et al., 1996). The eiders sampled in Newfoundland in this study were infected with *Gymnophallus* spp., which they could have acquired by eating blue mussels, although, the level of infection were less than in the eiders sampled in Greenland. Goudie and Ankney (1986) also found a range of gastropods, including *Littorina obtusata*, in the stomachs of eiders, supporting the presence of microphallid trematodes found in this study. The considerable infection levels of *Profilicollis* sp. and *Lateriporus* sp. in the eiders sampled in Newfoundland, suggest that these birds feed on crustaceans. However, Goudie and Ankney (1986) did not report any presence of amphipods in common eiders from Newfoundland and found only one decapod, *H. araneus*, in a low quantity, which does not support the high level of *Profilicollis* sp. infection found here, nor the results from Bishop and Threlfall (1974) reporting high prevalence and abundance of *P. botulus* in eiders caught in Newfoundland and Labrador. A possible explanation could be that the infection level of *Profilicollis* sp. in *H. araneus* is very high locally, whereby the probability of eiders getting infected is high despite *H. araneus* constituting a small percentage of the eider diet. Another explanation for this outcome is that some of the gastropod shells reported from Goudie and Ankney (1986) were inhabited by hermit crabs infected with *Profilicollis*, e.g. *P. pubescens*, and that any crab remains were too degraded to be identified. In contrast, Khan et al. (2011) found blue mussels and unidentified crustaceans in the digestive tracts of common eiders wintering in Newfoundland. In order to explain the difference in the infection levels from their study compared to Bishop and Threlfall (1974), the authors suggest that climate change may have caused a shift in the benthic community, and hence food resources for the common eiders. Such a shift could also explain the differences in the food items detected by Khan et al. (2011) and Goudie and Ankney (1986).

The discussion of the correlation between diet and intestinal parasites provided in this section suggests that the local habitat, and thereby the local benthic invertebrate community, is an important factor influencing infection levels in eiders. This is also corroborated by the result that the two subspecies sampled in Newfoundland did not differ in their parasitic load. More studies investigating the correlation between local intermediate host populations, diet, and parasitic infections in eiders, possibly including molecular tools, are needed in order to explore this issue further.

### 4.4 Body size, sex and age related differences

The results on the relationship between body size and parasite richness were ambiguous, since the nature of the correlation (if any) changed with location. Also, weak positive correlations between body size and parasite abundance were only found for eiders sampled in Cape Dorset, where larger eiders were infected with more individuals of *Lateriporus* sp., *Fimbriarioides* sp. and *Corynosoma* sp. than smaller eiders. Thus, our results do not directly support the expectation that larger hosts support a higher richness or abundance of parasites (Poulin, 1997), although it has recently been found in a meta-study on helminths in charadriiform birds (Gutiérrez et al., 2017).

No effects of age on parasitic load were detected in the eiders sampled in Greenland. This is in contrast to previous studies that find higher prevalences, abundances, and intensities in subadults compared to adults for *Profilicollis botulus* (Camphuysen et al., 2002; Thieltges et al., 2006; Thompson, 1985). However, Tourangeau et al. (2018) found that subadults had higher prevalence of *Profilicollis* sp. in only one of the three sampling years, and Skirnisson (2015) found no difference between *P. botulus* infections in adult and subadult eiders from Iceland. Given the small sample size of subadult individuals in this study, we suggest that our interpretation of age having no effect on gastrointestinal helminth infections in eiders should be treated with caution until additional research can corroborate these results.

We found no supported effect of sex on the intensities of the parasitic helminths in any of the eider groups, although male eiders sampled from Cape Dorset had a higher prevalence of the cestodes *Lateriporus* sp. and *Fimbriarioides* sp. compared to females. This is somewhat contrary to the findings in Provencher et al. (2016), where host sex did not have any effect on *Lateriporus* sp., although the infection intensity of *Profilicollis* sp. varied indirectly with sex through body condition, with females showing a higher infection intensity. Given that Provencher et al. (2016) was based on eiders from 2011 and here we also include eiders from Cape Dorset from 2012, this suggests that interannual differences may have influenced our results and that year and sex may have significant interactive effects. Females sampled in Greenland had a higher prevalence of *Microphallus* spp. compared to males, which could be due to females eating a higher proportion of periwinkles, the intermediate hosts of many microphallids, compared to males, although this pattern is not usually observed in spring, but rather during brood-care (Skirnisson, 2015). Another potential explanation is that males forage less during spring compared to females, due to courtship behavior (Steenweg et al., 2019) and thus acquire fewer infections. Determining resource use by birds of different sex, age and breeding activity is beyond the scope of this work, however, future studies could aim at including this aspect when examining parasitic infection levels in birds.

## 5 Conclusions

When studying species with wide ranges and distinct populations or subspecies, care must be taken in generalizing results from only one part of the range or one subspecies. This study demonstrates the variability of the parasitic load in a highly related group of marine birds. In resident common eiders, Tourangeau et al. (2018) found that sampling year, sex and age all varied significantly with some helminths, but not others. Their findings suggest that parasites in eider ducks may have complex distribution patterns that are unique to parasite species. This paper draws similar conclusions, and suggests that a factor such as prey distribution could be better predictor for the observed variation of parasitic infections compared to migration. Understanding levels of parasitic infections is pivotal in order to understand the health of host populations and more studies are needed to understand how the infection level of gastrointestinal helminths in eiders changes over time, especially regarding diet and prey populations as well as changes in climate. Climate change has already caused range shifts for several species and hence resulted in a ‘borealization’ of the Arctic, i.e. colonization of boreal species into the Arctic regions (e.g. Galaktionov, 2017; Josefson and Mokievsky, 2013; Kędra et al., 2015), which might result in increased availability of intermediate hosts, and thus potentially an increase the abundance of parasites that are able to infect eiders.

## Declaration of interest

None.
